# Optic Coherence Tomography Angiography Findings of Bilateral Choroidal Neovascularization Associated with Optic Disc Drusen Treated with Intravitreal Aflibercept Injection

**DOI:** 10.1155/2020/8398054

**Published:** 2020-03-10

**Authors:** Melih Akıdan, Mehmet Bulut, Lütfiye Yaprak, Muhammet Kazım Erol, Elcin Suren

**Affiliations:** ^1^Department of Ophthalmology, Antalya Kepez State Hospital, Antalya, Turkey; ^2^Department of Ophtalmology, Antalya Education and Research Hospital, Antalya, Turkey

## Abstract

**Purpose:**

The purpose of this case report is to present the findings of optical coherence tomography angiography (OCTA) of a patient with bilateral choroidal neovascularization (CNV) associated with optic disc drusen (ODD), who was treated with intravitreal aflibercept injection. *Case presentation.* A 14-year-old girl presented with a complaint of visual loss and metamorphopsia in her both eyes. Best-corrected visual acuity (BCVA) was 20/32 and 20/25, respectively, in the right and left eyes. Intraocular pressure and anterior segment examination were normal. Dilated fundus examination revealed elevated optic discs with blurred margins in both eyes. In addition, slightly elevated yellow lesion extending from optic nerve head to the macula was observed bilaterally. The patient underwent imaging with colour fundus photography, fundus autofluorescence (FAF), fundus fluorescein angiography (FFA), spectral-domain optic coherence tomography (SD-OCT), OCTA, orbital ultrasonography (USG), and computed tomography (CT). In particular, OCTA demonstrated clearly the large circular CNV complex in the right eye and the CNV structure in the left eye containing slightly activated main trunk and minimal vessel loops in the papillomacular region. CNV secondary to bilateral ODD was suspected. Intravitreal aflibercept injections were performed in 3 doses to the right eye and a single dose to the left eye. After the injections, BCVA reached its complete level in both eyes. SD-OCT revealed irregularity of RPE in the temporal region of the optic disc and complete regression of the subretinal fluid. Interestingly, the entire CNV complex including the main trunk completely disappeared in OCTA. CNV complex was not observed in OCTA during 1-year follow-up, and peripapillary and macular vascular density measurements did not show any significant change. BCVA was preserved, and no additional injections were needed.

**Conclusion:**

It is possible that OCTA can be used for detailed evaluation of CNV associated with ODD, response to anti-VEGF treatment, and peripapillary and macular vascular density. There is a need for further studies to confirm the changes such as disappearance of CNV in OCTA after injection as we observed in our patient.

## 1. Introduction

Optic disc drusen (ODD) are calcified hyaline-like deposits in the optic nerve head that are mainly located in front of the lamina cribrosa. They are often bilateral (67%-91%), and their incidence is 0.4%-20.4% in general population while there is female preponderance in their prevalence. They are usually benign and vision sparing; however, they may rarely complicate with visual field defects, haemorrhages, choroidal neovascularization (CNV), serous maculopathy, vascular occlusion, and nonarteritic ischemic optic neuropathy (NAION) [1].

Various imaging methods have been used in order to detect ODD accurately and reliably, which include ultrasonography (USG), enhanced depth imaging-OCT (EDI-OCT), fluorescein fundus angiography (FFA), and fundus autofluorescence (FAF), and the newest application is optical coherence tomography angiography (OCTA, Optovue, Inc., Fremont, CA, USA) [2].

Noninvasive imaging of the retina, choroidal, and disc microvasculature is now possible with OCTA and without the use of exogenous intravenous dye injection. It can provide information for the evaluation of both structure and blood flow. It particularly allows the assessment of CNV morphology and features activity as well as the timing of and response to anti-VEGF treatment of CNV [3, 4]. It has been increasingly used for the diagnosis and imaging of paediatric retinal vascular disease and especially CNV as it is both reliable and noninvasive [5].

The OCTA studies in the literature mainly focus on microvascular changes associated with optic disc drusen. To the best of our knowledge, only Ong et al. demonstrated inactivate CNV associated with ODD in 1 case with OCTA in a series of 8 paediatric patients with CNV. However, this is the first time to report an interesting change in CNV after injection and long-term follow-up findings.

## 2. Case Report

**Figure 1:**
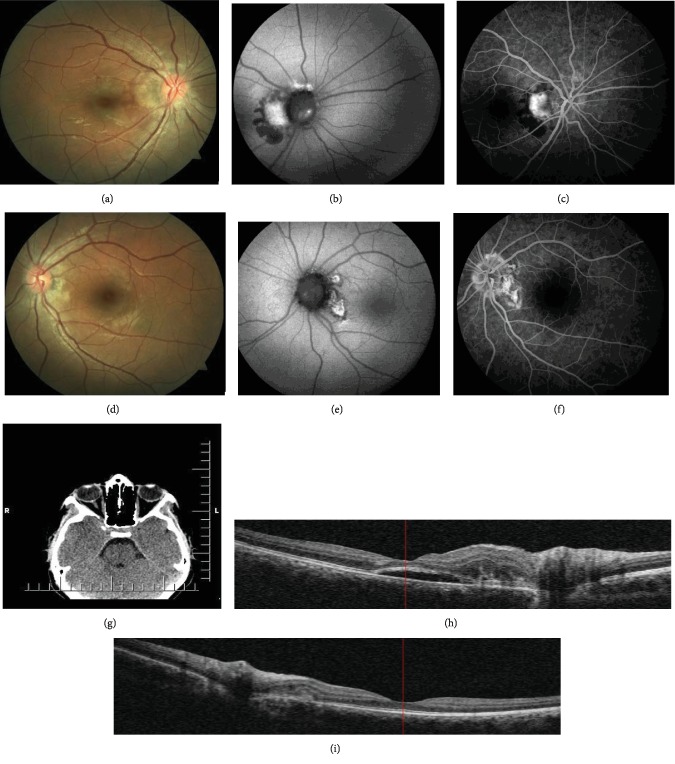
On the first day. (a and d) Fundus image of the right and left eyes; elevated optic disc with blurred margin and slightly elevated yellow lesion extending from optic nerve head to the macula. (b and e) FAF image of the right and left eyes; hyperautofluorescence bodies on the surface of the optic nerve linked with the drusen and hyperautofluorescent lesion in the papillomacular region. (c and f) FFA image of the right and left eyes; hyperfluorescence and intense leakage that might be associated with CNV. (g) Orbital CT image of the right and left eyes; bright spot on papilla associated with optic disc drusen. (h) SD-OCT image of the right eye; retinal thickening, subretinal hyperreflectivity, and subfoveal fluid in the papillomacular region. (i) SD-OCT image of the left eye; retinal thickening and subretinal hyperreflectivity in the papillomacular region.

A 14-year-old girl was referred to us with complaints of visual loss and metamorphopsia in both eyes that had persisted for 1 week. Her best-corrected visual acuity (BCVA) was 20/32 in the right eye and 20/25 in the left eye. Her ocular and systemic history was unremarkable. Her intraocular pressure and anterior segment examination were normal. Dilated fundus examination revealed elevated optic discs with blurred margins in both eyes. In addition, slightly elevated yellow lesion extending from optic nerve head to the macula was observed bilaterally (Figures 1(a) and 1(d)). The patient underwent imaging with colour fundus photography, FAF, FFA, spectral-domain optic coherence tomography (SD-OCT, Cirrus HD OCT 5000, Carl Zeiss Meditec Inc., Dublin, CA, USA), OCTA, orbital USG, and computed tomography (CT). FAF showed bilateral aspect of white refractile and hyperautofluorescence bodies on the surface of the optic nerve linked with the drusen and a hypoautofluorescent and hyperautofluorescent lesion in the papillomacular region (Figures 1(b) and 1(e)). FFA showed no hyperfluorescence or leakage with blurred borders of the optic disc, and early hyperfluorescence and intense leakage that might be associated with CNV were observed in both eyes with the right eye predominance (Figures 1(c) and 1(f)). The peripheral retina was normal. B-mode USG showed hyperechogenic appearance, and orbital CT revealed papilla as bright spots which might be associated with optic disc drusen (Figure 1(g)). SD-OCT showed retinal thickening, subretinal hyperreflectivity in the papillomacular region bilaterally, and subfoveal fluid in the right eye (Figures 1(h) and 1(i)). Furthermore, macular and retinal nerve fiber layer (RNFL) thickness measurements were recorded (Table 1).

**Figure 2:**
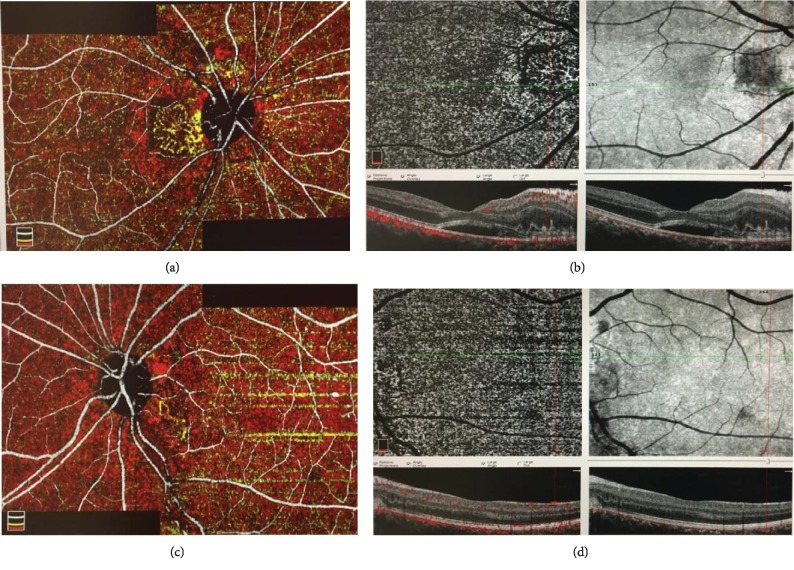
On the first day. (a) Unified and coloured OCTA image of the right eye; circular CNV complex with main trunk, multiple dense thin capillaries branching from the main trunk and frequent anastomoses (yellow). (b) Outer retina cross-section and en face OCTA image of the right eye. (c) Unified and coloured OCTA image of the left eye; CNV with main trunk, minimal vessel loops, and capillaries (yellow). (d) Outer retina cross-section and en face OCTA image of the left eye.

OCTA revealed superficial and deep capillary density, foveal avascular zone, flow, and en face images in the macula (Table 2). A large circular CNV complex with main trunk, multiple dense thin capillaries branching from the main trunk in a tree-like manner, and frequent anastomoses was observed in the papillomacular region of the right eye in the outer retina and choriocapillaris cross-sections (Figures 2(a) and 2(b)). In the left eye, CNV with main trunk, minimal vessel loops, and capillaries was observed, which was considered as slightly activated (Figures 2(c) and 2(d)). Moreover, radial papillary capillary density was measured with OCTA (Table 2). Density was lower in the areas associated with nasal quadrant compared to the other quadrants.

**Figure 3:**
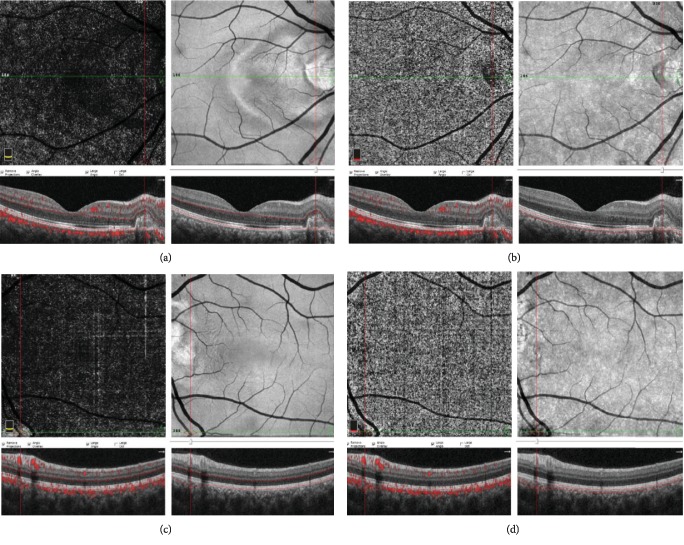
At 3 months. (a and b) Outer retina, choriocapillaris cross-sections, and en face OCTA image of the right eye; CNV complex including the main trunk disappearance. (c and d) Outer retina, choriocapillaris cross-sections, and en face OCTA image of the left eye; entire CNV compete of including the main trunk disappearance.

All evaluations suggested CNV secondary to ODD, and intravitreal aflibercept was injected in 3 doses to the right eye and a single dose to the left eye. Three doses were injected to the right eye every other month. After the first injection, BCVA remained as 20/32 and increased to 20/25 after the second one. After the third dose of injection to the right eye and the first one to the left eye, BCVA was 20/20 in both eyes. After the injections in both eyes, SD-OCT revealed RPE irregularity in the temporal region of the optic disc and complete regression of the subretinal fluid. OCTA interestingly showed that the entire CNV compete including the main trunk disappeared (Figures 3(a)to 3(d)). CNV complex was not observed with OCTA, and no significant change was observed in the peripapillary and macular vascular density measurements, and findings which might be correlated with RNFL measurements were not detected in 1-year follow-up. BCVA was preserved, and no additional injections were needed.

## 3. Discussion

CNV associated with ODD is a very rare complication, which has been reported in both adults and children [6]. In most of the patients, CNV membranes associated with ODD occur nasally; however, they may also occur temporally resulting in serous haemorrhagic maculopathy, cystoid macular edema, and macular scarring [7]. Its pathogenesis is unknown. Possible mechanisms involved in this complication include the compressive effect of drusen on the surrounding blood vessels, which leads to mechanical impairment of peripapillary vascular integrity, vascular congestion, or ischemia. Retinal ischemia and the release of VEGF may be the factors that trigger the development of CNV [8, 9]. Treatment must be provided if vision is jeopardized. The modern antivascular endothelial growth factor (VEGF) medications, photodynamic therapy, and surgical removal have each been demonstrated to be successful [10]. Anti-VEGF agents have been increasingly preferred as the first-line treatment because they ensure high visual acuity and disease stabilization usually with fewer intravitreal injections especially in paediatric cases, and recurrence is almost never observed. Gan and Long reported complete resolution after 3 doses of injection in cases for whom aflibercept was injected for the first time [11]. Knape et al. reported rapid and atraumatic resolution with the combination of bevacizumab and focal laser photocoagulation [12]. Alkın et al. emphasized that CNV secondary to ODD might be more sensitive than CNV secondary to age-related macular degeneration (AMD) in cases injected with a single dose of ranibizumab [13]. We achieved rapid response and did not observe recurrence in cases injected with 3 doses of aflibercept in the right eye and a single dose of aflibercept in the left eye and whom we followed for 12 months with OCTA.

4 different CNV patterns associated with exudative age-related macular degeneration can be detected with OCTA: Medusa pattern: central feeder vessel, circular peripheral anastomosis, thin branches, and hypointense halo; seafan pattern: eccentric feeder vessel, thin branches, and hypointense halo; indistinct network pattern: thin branches and hypointense halo; and pruned vascular tree pattern: persistence of main vascular trunks [14]. Pruned vascular tree pattern is considered as inactivate and usually followed up. The morphology of CNV and structural response to anti-VEGF treatment are strongly correlated. Anti-VEGF is ineffective for the vascular trunk while it is effective for especially the newly formed capillaries from preexisting vascular trunks while there is a correlation between better functional outcomes an increased vascularity of the CNV [15]. Spaide demonstrates reprofileration 20-50 days after the injection [16]. However, long-term inactivation can be ensured with fewer anti-VEGF injections in cases of CNV associated with high myopia, punctate inner choroidopathy (PIC), choroidal osteoma, optic nerve drusen like our patient, and idiopathic with lower activity than AMD [17–20]. The literature does not contain wide range of data which can be used to assess why stabilization can be achieved with fewer injections and make comparisons with CNV patterns of AMD with OCTA as highlighted above and assess reproliferation.

The most striking finding after injection in our case was the absence of CNV visualization in OCTA. The absence of CNV visualization in OCTA was associated with clinical and SD-OCT exudative inactivity [14]. In fact, inactive lesions are visualized as pruned vascular tree pattern, presence of large linear vessel with no or minimal anastomosis in OCTA. This may be due to other reasons such as the masking effect generated by a high PED, media opacities, or the presence of haemorrhages [21]. However, they were not observed in the follow-up of our patient. Hua and Ning demonstrated the development of atrophy in the CNV complex after 3 doses of anti-VEGF injections in PIC cases with high myopia and round dark region visualization due to no-perfusion/hypopefusion zone in the choriocapillaris [22]. This situation which was also observed in our case can be interpreted as decreased signal due to inactivity, but the absence of pruned vascular tree pattern may at least contribute to explaining why atrophy/shrinkage can develop in the main vascular trunk and why non-AMD CNV causes are more benign and can be stabilized longer with fewer injections.

In the literature, only Ong et al. demonstrated inactive CNV associated with ODD in 1 case with OCTA in a series of 8 paediatric patients with CNV. A 12-year-old female patient first underwent injection and photodynamic therapy in the left eye; however, OCTA revealed two large calibre vessels and a lack of fine capillaries, anastomoses, and vessel loops, which was considered as inactive sub-RPE CNV lesion [23]. This is the first paper to report a case with an interesting change in CNV visualization after injection and followed up for a long term for both CNV and vascular density. On the other hand, studies on OCTA for ODD mainly focus on microvascular changes. Biçer and Atilla showed that macular vascular density located in different regions decreased both at the superficial and deep capillary layer in cases with bilateral ODD, and there was a density loss in the peripapillary area especially the nasal region. They reported that ODD findings could be used to demonstrate that enlarged ODD might cause acute or chronic ischemia by compressing nerve fibers or surrounding vessel [24]. Aghdam et al. compared patients with ODD, NAION, and normal individuals and reported that optic nerve head vessel density was lower in the NAION group than in the other groups [25]. Cennamo et al. reported that flow rate measurements were correlated with ganglion cell layer thickness and OCTA examination can be an early marker to show the axonal damage in patients with ODD [26]. Similarly, Engelke et al. demonstrated the correlation between vascular density and RNFL and ganglion cell complex (GCC) and peripapillary capillary density loss compared to the normal group [27]. In our case, however, peripapillary capillary density evaluation revealed that the vascular density in the nasal quadrants was lower than those in the other quadrants, which was an interesting finding. However, changes that might be correlated in both RNFL and disc and macular vascular density measurements were not detected contrary to the abovementioned reports.

In conclusion, it is possible to evaluate vascular density, presence of associated CNV, and response to injection with OCTA in patients with optic nerve diseases such as ODD. Nonetheless, there is a need for further studies to confirm the changes in CNV visualization like in our case with OCTA that has been increasingly used.

## Figures and Tables

**Table 1:** On the first day, 3rd month, and 12th month; OCT and RNFL thickness measurements.

Thickness (*μ*m)	Day 1		Month 3		Month 12	
*Macular*						
Fovea	232	250	236	248	239	251
Parafovea	337	340	339	335	338	348
Superior-hemi	339	342	340	338	339	350
Inferior-hemi	335	337	337	333	336	345
Temporal	323	319	328	316	327	328
Superior	348	349	349	344	349	358
Nasal	335	350	335	347	332	354
Inferior	343	340	344	334	343	350
Perifovea	318	322	316	316	316	325
Superior-hemi	322	328	317	322	315	330
Inferior-hemi	315	316	314	310	316	320
Temporal	293	296	299	292	297	302
Superior	322	330	320	323	318	333
Nasal	341	349	330	342	329	346
Inferior	317	313	314	307	318	320
*Retinal nerve fiber layer*						
Whole	129	126	126	125	129	134
Superior-hemi	123	130	121	129	122	142
Inferior-hemi	137	122	131	121	136	126
Nasal superior	111	107	116	102	117	110
Nasal inferior	107	97	100	91	108	98
Inferior nasal	145	146	142	141	149	147
Inferior temporal	195	173	202	182	211	184
Temporal inferior	108	74	87	78	82	80
Temporal superior	100	97	88	100	91	107
Superior temporal	176	179	173	185	174	201
Superior nasal	118	157	119	150	117	174

Cells are divided into two to show the results of the right and left eyes separately.

**Table 2:** On the first day, 3rd month, and 12th month; OCTA macula and disc vascular density, FAZ, and flow measurements.

	Day 1		Month 3		Month 12	
Disc-capillary density						
Radial peripapillary (%)						
Whole	49.1	48.6	49.2	48.1	48.2	48.5
Inside disc	46.9	45.3	45.9	43.3	40.6	45.9
Peripapillary	51.7	51.0	51.1	49.5	50.7	50.4
Superior-hemi	50.7	50.8	51.2	49.9	50.7	50.5
Inferior-hemi	52.8	51.2	50.9	49	50.7	50.2
Nasal superior	45.8	47.8	46	47.1	46.1	46.3
Nasal inferior	49.1	46.6	48.4	46.6	49.6	46.3
Inferior nasal	53.1	50.9	52.3	45.4	51.8	48.3
Inferior temporal	53.8	57.2	52.3	53.1	49.7	57.2
Temporal inferior	56	51.5	51.4	52.2	51.7	51.1
Temporal superior	57.3	55.3	57.1	56	56.7	58.2
Superior temporal	56.1	53.2	55.9	53.3	52.4	50.9
Superior nasal	45.7	47.9	47.2	43.9	48.2	47.7
Macula-capillary density						
Superficial (%)						
Whole	52.4	53.2	54.5	54.1	53.5	51.9
Superior-hemi	53.5	53	54.8	53.7	53.3	51.9
Inferior-hemi	54.6	50.8	54.2	54.5	53.6	51.9
Fovea	15.2	17.1	14.6	17.7	15.7	15.3
Parafovea	55.3	53.6	58.2	58.1	56.8	55
Superior-hemi	57.1	53.5	57.8	56.6	55.9	54.1
Inferior-hemi	56.8	56.8	58.6	59.6	57.7	56
Temporal	56.2	52.8	55.5	56.5	56.1	54.1
Superior	58.5	55.6	58.8	57.4	57.2	53.6
Nasal	56.7	59.3	57.5	57.9	55.4	55.6
Inferior	60	55.9	61	60.5	58.4	56.8
Perifovea	52.8	51.8	54.3	54.5	53.3	52.9
Superior-hemi	53.1	53.5	54.7	54.3	52.9	53.2
Inferior-hemi	53.6	54.1	53.9	54.7	53.6	52.7
Temporal	50.3	52	51.8	52.2	50.8	52.5
Superior	54.2	54.7	54.7	53.9	52.4	53.1
Nasal	57.5	53.4	57.7	58.2	57.4	55.2
Inferior	53.4	52.4	52.6	53.8	52.2	50.9
Deep (%)						
Whole	48.1	47.6	48.3	48.4	48	46.9
Superior-hemi	49.9	47.9	50.3	47.9	49.7	47.4
Inferior-hemi	46.4	48.5	46.4	49	46	46.4
Fovea	32.3	30.1	30.6	29.1	32.7	29.2
Parafovea	55.8	53.9	56.4	55.1	55.5	55.7
Superior-hemi	56.7	55.7	56.7	55.2	56.2	55.9
Inferior-hemi	55.6	56.2	56	54.9	54.8	55.5
Temporal	55.8	57.5	55	57.4	55.6	57.8
Superior	56.5	53.4	57.8	53.7	55.5	55.3
Nasal	57.9	55.2	58.3	55.9	58.1	55.8
Inferior	52.3	51.8	54.4	53.4	52.8	53.9
Perifovea	50.1	48.3	50.7	50.1	48.1	46.3
Superior-hemi	50.2	48.6	52.8	49.8	49	46.7
Inferior-hemi	48.5	47.4	48.6	50.4	47.2	45.8
Temporal	54.8	54.3	56.1	54.6	55.5	49.5
Superior	49.4	49.2	53.3	49.4	47.3	47.4
Nasal	43.9	43.4	46.4	46.5	44.6	42.8
Inferior	46.7	48.1	47.8	50	45.8	45.3
Foveal avascular zone (mm^2^)						
Retina	352	376	347	389	358	386
Foveal flow						
Outer retina	0.558	0.732	0.346	0.846	0.602	0.665
Choriocapillaris	2.101	2.068	2.087	2.091	2.195	2.058

Cells are divided into two to show the results of the right and left eyes separately.

## References

[1] Padhy K. S., Behera U. C. (2019). Optic disc drusen precipitating central retinal vein occlusion in young. *BMJ Case Reports*.

[2] Gise R., Gaier E. D., Heidary G. (2019). Diagnosis and imaging of optic nerve head drusen. *Seminars in Ophthalmology*.

[3] Lumbroso B., Rispoli M., Savastano M. C., Jia Y., Tan O., Huang D. (2016). Optical coherence tomography angiography study of choroidal neovascularization early response after treatment. *Developments in Ophthalmology*.

[4] Wang R., Liang Z., Liu X. (2019). Diagnostic accuracy of optical coherence tomography angiography for choroidal neovascularization: a systematic review and meta-analysis. *BMC Ophthalmology*.

[5] Veronese C., Maiolo C., Huang D. (2016). Optical coherence tomography angiography in pediatric choroidal neovascularization. *American Journal of Ophthalmology Case Reports*.

[6] Saffra N. A., Reinherz B. J. (2015). Peripapillary choroidal neovascularization associated with optic nerve head drusen treated with anti-vegf agents. *Case Reports in Ophthalmology*.

[7] Rotruck J. (2018). A review of optic disc drusen in children. *International Ophthalmology Clinics*.

[8] Delas B., Almudi L., Asaad M. (2012). Bilateral choroidal neovascularization associated with optic nerve head drusen treated by antivascular endothelial growth factor therapy. *Clinical Ophthalmology*.

[9] Law D. Z., Yang F. P., Teoh S. C. (2014). Case report of optic disc drusen with simultaneous peripapillary subretinal hemorrhage and central retinal vein occlusion. *Case Reports in Ophthalmological Medicine*.

[10] Palmer E., Gale J., Crowston J. G., Wells A. P. (2018). Optic nerve head drusen: an update. *Neuro-Ophthalmology*.

[11] Gan W. L., Long V. W. (2019). Paediatric case of peripapillary choroidal neovascularisation associated with optic disc drusen treated with aflibercept. *BMJ Case Reports*.

[12] Knape R. M., Zavaleta E. M., Clark C. L., Khuddus N., Peden M. C. (2011). Intravitreal bevacizumab treatment of bilateral peripapillary choroidal neovascularization from optic nerve head drusen. *Journal of American Association for Pediatric Ophthalmology and Strabismus*.

[13] Alkın Z., Ozkaya A., Yılmaz I., Yazici A. T. (2014). A single injection of intravitreal ranibizumab in the treatment of choroidal neovascularisation secondary to optic nerve head drusen in a child. *Case Reports*.

[14] Miere A., Butori P., Cohen S. Y. (2019). Vascular remodeling of choroidal neovascularization after anti-vascular endothelial growth factor therapy visualized on optical coherence tomography angiography. *Retina*.

[15] Ichiyama Y., Sawada T., Ito Y., Kakinoki M., Ohji M. (2017). Optical coherence tomography angiography reveals blood flow in choroidal neovascular membrane in remission phase of neovascular age-related macular degeneration. *Retina*.

[16] Spaide R. F. (2015). Optical coherence tomography angiography signs of vascular abnormalization with antiangiogenic therapy for choroidal neovascularization. *American Journal of Ophthalmology*.

[17] Traversi C., Nuti E., Marigliani D. (2015). Forty-two-month outcome of intravitreal bevacizumab in myopic choroidal neovascularization. *Graefe's Archive for Clinical and Experimental Ophthalmology*.

[18] Arrevola L., Acero M. A., Peral M. J. (2019). Two-year outcome of aflibercept for the treatment of choroidal neovascularization in punctate inner choroidopathy. *Case Reports in Ophthalmological Medicine*.

[19] Chen Q., Yu X., Sun Z., Dai H. (2016). The application of OCTA in assessment of anti-VEGF therapy for idiopathic choroidal neovascularization. *Journal of Ophthalmology*.

[20] Szelog J. T., Bonini Filho M. A., Lally D. R., de Carlo T. E., Duker J. S. (2016). Optical coherence tomography angiography for detecting choroidal neovascularization secondary to choroidal osteoma. *Ophthalmic Surgery, Lasers and Imaging Retina*.

[21] Spaide R. F., Fujimoto J. G., Waheed N. K. (2015). Image artifacts in optical coherence tomography angiography. *Retina*.

[22] Hua R., Ning H. (2019). Using optical coherence tomography angiography to guide the treatment of pathological myopic patients with submacular haemorrhage. *Photodiagnosis and Photodynamic Therapy*.

[23] Ong S. S., Hsu S. T., Grewal D. (2020). Appearance of pediatric choroidal neovascular membranes on optical coherence tomography angiography. *Graefe's Archive for Clinical and Experimental Ophthalmology*.

[24] Biçer O., Atilla H. (2019). Microvascular changes associated with optic disc drusen: case report. *Turkish Journal of Ophthalmology*.

[25] Aghdam K. A., Khorasani M. A., Sanjari M. S. (2019). Optical coherence tomography angiography features of optic nerve head drusen and nonarteritic anterior ischemic optic neuropathy. *Canadian Journal of Ophthalmology*.

[26] Cennamo G., Tebaldi S., Amoroso F., Arvanitis D., Breve M., Cennamo G. (2018). Optical coherence tomography angiography in optic nerve drusen. *Ophthalmic Research*.

[27] Engelke H., Shajari M., Riedel J., Mohr N., Priglinger S. G., Mackert M. J. (2019). OCT angiography in optic disc drusen: comparison with structural and functional parameters. *British Journal Ophthalmology*.

